# Nasal Asthma and Emphysema

**Published:** 1898-02

**Authors:** 


### NASAL ASTHMA AND EMPHYSEMA.

According to the Louisville Medical Monthly Dr. Stech lays down the rule that bronchial spasm is the first, bronchitis the second, and over-distention of the lungs the third manifestation of the asthmatic attack. This over-distention of the lung disappears more or less rapidly, so that there can be no question of emphysema in the anatomical sense. The asthmatic attack may be indefinitely prolonged, so that the dyspnœa persists, and is aggravated at night. The author says that these patients when advanced in life are regarded as the subjects of emphysema, and are so treated, and yet profound changes may be present in the nose, of which not even the patient himself is aware. Here, after the removal of polypic etc., the condition may be improved, but never perfectly cured. The author would conclude that after asthmatic attacks lasting for years emphysema in the anatomical sense may occur in elderly people, but never within his experience in young subjects. The dyspnœa of emphysema is too often attributed to asthma. While bronchial asthma of nasal origin occurs when the patient is at rest, and especially at night, the dyspnœa of emphysema mostly appears on exertion. The author maintains that in nasal asthma the bronchial asthma may even occur on one side only. Nasal asthma may appear in an incomplete form, and is often hidden under the appearance of a chronic bronchitis. The author would look upon this asthma as a neurosis, as its rapid disappearance after nasal treatment would show.

